# Leaf phosphorus content of *Quercus wutaishanica* increases with total soil potassium in the Loess Plateau

**DOI:** 10.1371/journal.pone.0201350

**Published:** 2018-08-02

**Authors:** Kaixiong Xing, Mingfei Zhao, Chen Chen, Yuhang Wang, Feng Xue, Yiping Zhang, Xiaobin Dong, Yuan Jiang, Han Y. H. Chen, Muyi Kang

**Affiliations:** 1 State Key Laboratory of Earth Surface Processes and Resource Ecology, Beijing Normal University, Haidian District, Beijing, China; 2 College of Resources Science & Technology, Beijing Normal University, Haidian District, Beijing, China; 3 College of Life Sciences, Beijing Normal University, Haidian District, Beijing, China; 4 Faculty of Natural Resources Management, Lakehead University, Thunder Bay, Ontario, Canada; Helmholtz Centre for Environmental Research - UFZ, GERMANY

## Abstract

Phosphorus (P) is arguably more limiting than nitrogen for forest ecosystems being free of disturbances for lengthy time periods. The elucidation of multivariate relationships between foliar P and its primary drivers for dominant species is an urgent issue and formidable challenge for ecologists. Our goal was to evaluate the effects of primary drivers on foliar P of *Quercus wutaishanica*, the dominant species in broadleaved deciduous forest at the Loess Plateau, China. We sampled the leaves of 90 *Q*. *wutaishanica* individuals across broad climate and soil nutrient gradients at the Loess Plateau, China, and employed structural equation models (SEM) to evaluate multiple causal pathways and the relative importance of the drivers for foliar P per unit mass (P_mass_) and per unit area (P_area_). Our SEMs explained 73% and 81% of the variations in P_mass_ and P_area_, respectively. P_mass_ was negatively correlated to leaf mass per area, positively correlated to leaf area, and increased with mean annual precipitation and total soil potassium. P_area_ was positively correlated to leaf mass per area, leaf dry weight, and increased significantly with total soil potassium. Our results demonstrated that leaf P content of *Q*. *wutaishanica* increased with total soil potassium in the Loess Plateau accordingly.

## Introduction

Leaf nitrogen (N) and phosphorus (P) play crucial roles in productivity and other biological processes [1–6]. In terrestrial ecosystems, N and P are the most common limiting elements, either individually, and/or in combination [7–11]. Nitrogen supply increases with on-going increases in atmospheric N deposition [12]. By contrast, a fixed complement of P is primarily derived from rock weathering, and even very small losses of P may not be readily replenished [13]. The availability of P declines during long-term ecosystem development, and may eventually lead to P-deficient soils [5, 7, 13]. Although N is frequently proposed as the primary limiting nutrient in forests (particularly in boreal and temperate biomes), P is arguably more limiting for systems that have been free of disturbances for lengthy time periods [11, 14].

This relative P limitation may be further intensified under continuous and widespread anthropogenic N emissions [11, 15]. High N deposition, typically from anthropogenic emissions, decreases soil C:N ratio and increases N supply to plants [12]. In China, which is currently, by far, the largest creator and emitter of anthropogenic N globally, foliar N was observed to increase significantly between 1980 and 2000 across all plant species; however, foliar P was not altered over the same time period [15]. As a vital element for energy storage and cell structure, P has emerged as a key limiting nutrient to plant development and growth, spanning from regional to global scales [1, 4, 16].

Leaf P per mass (P_mass_) and per unit area (P_area_) are two important indices for leaf P status, and their respective advantages remain under discussion. P_mass_ appeals more to researchers who are concerned with plant growth and economics, such as resource investment and return, whereas P_area_ has a greater appeal for researchers who are interested in photosynthetic physiology, which focuses on the implementation of leaf functions [17–19].

On an extensive regional or global scale, leaf resident N and P increases from the tropics to the cooler and drier mid-latitudes [7]. Moreover, the first global quantification of the relationship between leaf phosphorus status and soil nutrients indicated that leaf P was positively correlated to soil P; however, soil P was negatively associated with precipitation [20]. Thus, it remains unclear as to whether foliar P is determined by soil P or precipitation. Potassium (K) is the most abundant cation in plant cells and the second most abundant nutrient after N in leaves, but is a limited nutrient in 70% of all studied terrestrial ecosystems [21]. Soil K is critical to K supply for plants because the loss of K from leaves through leaching is more pronounced than for other elements [22, 23]. K deficiency reduced photosynthesis [4], and impaired phloem transport of sucrose to root systems, which are important for the fine root growth and absorption of mineral nutrients, including P [24–28]. We therefore hypothesize that soil resident K is positively associated with foliar P.

The intraspecific relationships between leaf morphological traits (LMT) and leaf P gradually got more attention in recent years, especially for widespread species. Leaf mass per area (LMA) quantifies the leaf dry-mass investment per unit of light-intercepting leaf area [29, 30]. The negative correlations between LMA and P_mass_ were observed in *Phragmites australis* and *Robinia pseudoacacia* [31, 32], which was coordinated with the relationships at interspecific scale [17, 31, 33, 34]. The limitation of P initiates reductions in leaf area (LA) and leaf growth due to both the direct effects of P shortage on leaf expansion rates, and the reduction of assimilation products that are required for growth [35, 36]. However, the associations between LA and leaf dry weight (LDW), which are independent of LMA [37–39], and foliar P remain poorly understood.

Recent literature has revealed that intraspecific variation in leaf traits is greater than previously supposed [40, 41]. To better understand the determinants of foliar P within species, we studied the P_mass_ and P_area_ of the Liaotung oak (*Quercus wutaishanica*), which is a dominant and widely distributed species of the temperate deciduous broad-leaved forests along natural gradients of climate and soil nutrient variability in the Loess Plateau, in Northern China [42]. Here, we tested following hypotheses that: (i) in addition to LMA, LA and LDW may also contribute to the variation of foliar P; (ii) the influences of environmental, especially for soil K, and leaf trait variables on P_mass_ and P_area_ differ in strength and even directions. By using structural equation modeling (SEM) [43], we examined the influences of climate, soil nutrients, and the morphological traits of leaves on the variations in leaf P_mass_ and P_area_ of *Q*. *wutaishanica*.

## Materials and methods

### Study area

This study was conducted on the Loess Plateau in Northern China, where *Q*. *wutaishanica* is primarily distributed. The 30 sites were distributed across six mountains, with the same locations as Xing, Kang (28) (Table 1 and S1 Table). The mean annual temperature (MAT) and mean annual precipitation (MAP) ranged from 4.1 to 10.3 °C, and 554 to 880 mm, respectively, whereas the elevation ranged from 1252 to 2303 m (Table 1). In the upper 0–20 cm soil layer, total soil N, P, K were on average 2.48 mg g^-1^, 0.52 mg g^-1^, and 19.57 mg g^-1^, respectively (Table 1).

**Table 1:** Description of sampling range, environmental conditions, and leaf traits.

Variables	Mean	SD	Minimum	Maximum	CV (%)
**Elevation (m)**	1700	--	1252	2303	--
**Longitude (°)**	--	--	106.68233	113.50182	--
**Latitude (°)**	--	--	34.04959	37.13302	--
**MAT (°C)**	6.7	1.84	4.1	10.3	27
**MAP (mm)**	637	94.47	554	889	15
**TSN (mg g**^**-1**^**)**	2.48	1.84	0.90	9.60	71
**TSK (mg g**^**-1**^**)**	19.57	2.56	14.10	25.90	13
**TSP (mg g**^**-1**^**)**	0.52	0.20	0.20	1.30	39
**LMA (g m**^**-2**^**)**	77.06	2.16	35.18	132.85	23
**LA (cm**^**2**^ **leaf**^**-1**^**)**	35.82	11.31	19.50	72.40	28
**LDW (g leaf**^**-1**^**)**	0.27	14.01	0.13	0.51	30
**N**_**mass**_ **(mg g**^**-1**^**)**	23.59	3.42	17.60	33.70	15
**N**_**area**_ **(g m**^**-2**^**)**	1.79	0.39	1.00	3.10	22
**P**_**mass**_ **(mg g**^**-1**^**)**	1.12	0.29	0.52	1.88	26
**P**_**area**_ **(g m**^**-2**^**)**	0.09	0.03	0.05	0.23	34
**K**_**mass**_ **(mg g**^**-1**^**)**	6.71	1.44	2.79	10.88	21
**K**_**area**_ **(g m**^**-2**^**)**	0.51	0.15	0.26	0.94	29
**N:P ratio**	21.78	0.54	13.15	41.76	20.8

Abbreviations: MAT, mean annual temperature; MAP, mean annual precipitation; TSN, total soil nitrogen; TSK, total soil potassium; TSP, total soil phosphorus; LMA, leaf mass per area; LA, leaf area; LDW, leaf dry weight; P_mass_, leaf phosphorus per unit mass; N_mass_, leaf nitrogen per unit mass; K_mass_, leaf potassium per unit mass; N_area_, leaf nitrogen per unit area; P_area_, leaf phosphorus per unit area; K_area_, leaf potassium per unit area; SD, standard deviation; CV, the coefficient of variation.

### Sample collection

To ensure a wide coverage of habitat conditions, we selected sample sites at every 100-m elevation interval on each of the six mountains, and a total of 90 samples from 30 sites were collected. Subsequent to confirming that every sample site was at least 50 m from any clearing, and 100 m from any roadway, we randomly sampled three healthy *Q*. *wutaishanica* trees, and collected 20 canopy leaves from the sun-exposed side of each tree. Within a 400 m^2^ vicinity, three soil samples were randomly collected from a 0–20 cm soil depth, where the bulk of the fine roots of most plants occur, and large amounts of N, P, and K accumulate, due to uplift and release at the surface by plants through litter fall and fine root turnover [44, 45]. Three soil samples were combined to make a composite sample from each sample site, yielding a total 30 composite soil samples for laboratory analysis [46].

### Variable measurements

Leaf nutrients and morphological traits were measured or calculated for each sampled *Q*. *wutaishanica* tree. We determined the average LA (cm^2^ leaf^-1^) of each tree by scanning the fresh leaves. Subsequent to oven drying these leaf samples at 80°C for 48 hours, we quantified the average LDW (g leaf^-1^), and LMA (g m^-2^) was calculated by LDW/LA. The oven-dried samples were then pulverized using a plant sample mill and sieved through a 0.15 mm mesh screen. We employed the CHNS element analyzer (Vario EL III, Elementar Analyser systeme GmbH, Hanau, Germany) to determine the N concentrations (N_mass_, mg g^-1^) of each sample. The P concentrations (P_mass_, mg/g) of each sample were quantified using inductively coupled plasma atomic emission spectrometry measurements (ICP-AES, SPECTRO ARCOS EOP, SPECTRO, Germany) after dissolved 65% nitric acid (HNO_3_). The P_area_ and N_area_ were calculated as P_mass_ and N_mass_ × LDW/LA, respectively [47].

Soil samples were oven-dried at 50°C to constant weight, pulverized using a soil sample mill, sieved through a 0.15 mm mesh screen, and then analyzed for total soil nitrogen (TSN) (mg g^-1^) via the CHNS element analyzer (Vario EL III, Elementar Analyser systeme GmbH, Hanau, Germany). Total soil P (TSP) and total soil K (TSK) were quantified using inductively coupled plasma atomic emission spectrometry measurements (ICP-AES, SPECTRO ARCOS EOP, SPECTRO, Germany) after microwave-digestion with for HCl-HNO_3_ (3:1 by volume) (Sandroni and Smith 2002).

Mean annual temperature (MAT) and mean annual precipitation (MAP) were extracted from the WorldClim spatial climate data (period from 1950 to 2000 with resolution at ca 1 km, available at www.worldclim.org/) based on spatial coordinates (latitude, longitude, and elevation). The spatial location, i.e., latitude, longitude, and elevation, of each sample tree was determined via GPSMAP 629sc (Garmin).

### Data analyses

**Fig 1:**
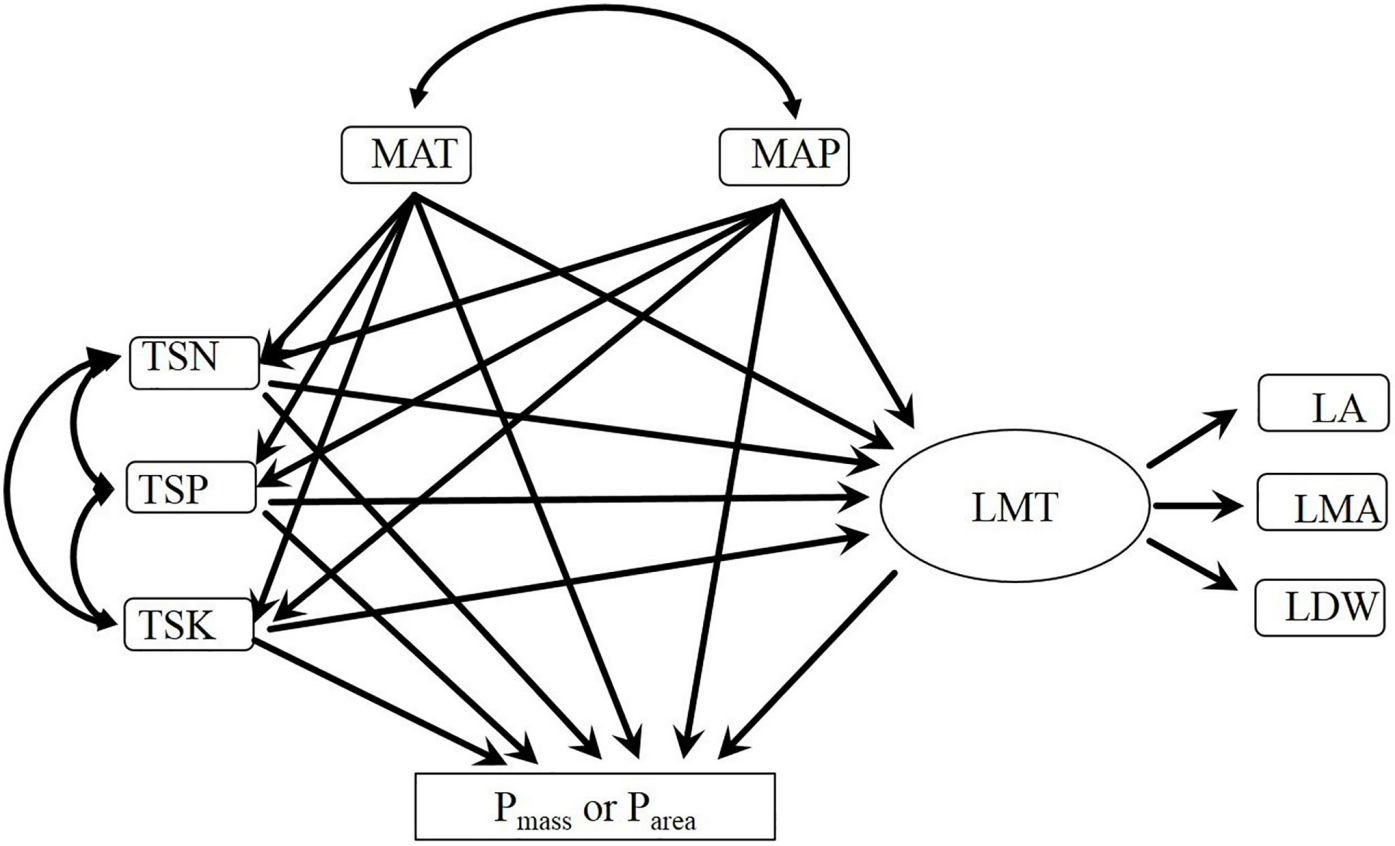
The *a priori* model for leaf P concentrations. Single headed arrows indicate a causal influence of one variable upon another. Double headed arrows indicate correlation between two variables. Abbreviations: MAT, mean annual temperature; MAP, mean annual precipitation; TSN, total soil nitrogen; TSK, total soil potassium; TSP, total soil phosphorus; LMT, leaf morphological trait; LMA, leaf mass per area; LA, leaf area; LDW, leaf dry weight; P_mass_, leaf phosphorus per unit mass; P_area_, leaf phosphorus per unit area.

Structural equation models (SEMs) have been increasingly employed in ecology to separate direct and indirect effects between exogenous and endogenous variables [48–50]. An integrative modeling approach has led to a major advance in the ability to discern underlying processes in ecological systems [51]. We first examined the bivariate correlation between variables, and then established an *a priori* model based on a known theoretical construct, according to the previous foliar P studies mentioned above, including key variables and possible paths (Table 1, Fig 1). LMA, LDW, and LA are pairwise correlated leaf morphological traits influencing leaf P. In order to represent the synthetic effects on foliar P, three traits were incorporated into leaf morphological trait (LMT), which was the theoretical concept that combined three manifest variables to a latent variable (Fig 1). Each of the three observable soil nutrient variables (TSN, TSP, and TSK) had its path to foliar P, due to their different roles in plant physiology and growth [52]. MAT and MAP also had separate paths to soil nutrients, LMT and foliar P (Fig 1). Subsequently, we employed stepwise procedures, guided by Akaike information criterion values, to obtain the most parsimonious set of predictors [48]. We adopted several indices to evaluate the suitability of the final models: the chi-square test (χ^2^), the root square mean error of approximation (RMSEA), the adjusted goodness of fit index (AGFI), the normed fit index (NFI) and χ^2^/d.f. (NC) [53]. SEM analyses were performed with AMOS 20.0 package, and correlation analyses were performed with IBM SPSS 20.0.0 (IBM SPSS, Inc., Chicago, IL, USA).

## Results

### Correlations between leaf P content, leaf morphological traits and environmental factors

P_mass_ was negatively correlated to LMA (Table 2) and positively correlated to LA (Table 2), and P_area_ was positively correlated to LMA and LDW (Table 2). Both P_mass_ and P_area_ increased caused by higher TSK (Table 2). There was also positive correlation between TSK and TSP (Table 2), and both of which decreased caused by higher MAP (Table 2). There were no clear relationships between MAT, TSN and leaf traits.

**Table 2:** Pearson correlation coefficients between variables.

	**P**_**mass**_	**P**_**area**_	**N**_**mass**_	**N**_**area**_	**K**_**mass**_	**K**_**area**_	**LMA**	**LDW**	**LA**	**TSN**	**TSK**	**TSP**	**MAT**
**P**_**area**_	0.63***												
**N**_**mass**_	0.61***	0.11											
**N**_**area**_	0.13	0.76***	0.06										
**K**_**mass**_	0.58***	0.25*	0.40**	-0.04									
**K**_**area**_	0.23	0.70	-0.13	0.68**	0.54**								
**LMA**	-0.26*	0.54***	-0.53***	0.74***	-0.23	0.63**							
**LDW**	0.22	0.50***	-0.03	0.45***	0.16	0.47**	0.38**						
**LA**	0.42***	0.05	0.39**	-0.17	0.38**	-0.03	-0.34**	0.69***					
**TSN**	-0.02	0.10	-0.23	0.05	0.07	0.17	0.15	0.09	-0.07				
**TSK**	0.37**	0.60***	0.18	0.55***	0.11	0.39**	0.35**	0.28*	0.03	-0.08			
**TSP**	0.03	0.23	-0.07	0.27*	0.13	0.13	0.30*	0.23	-0.01	0.03	0.58***		
**MAT**	-0.05	-0.03	-0.17	-0.04	0.03	0.11	0.06	-0.06	-0.11	-0.07	0.09	0.13	
**MAP**	0.03	-0.02	-0.21	-0.17	0.12	0.04	-0.03	-0.02	-0.03	0.59***	-0.53***	-0.36**	-0.34**

Significances are at *P* < 0.05 (*), < 0.01 (**), and < 0.001 (***). Abbreviations: MAT, mean annual temperature; MAP, mean annual precipitation; TSN, total soil nitrogen; TSK, total soil potassium; TSP, total soil phosphorus; LMA, leaf mass per area; LA, leaf area; LDW, leaf dry weight; N_mass_, leaf nitrogen per unit mass; N_area_, leaf nitrogen per unit area; P_mass_, leaf phosphorus per unit mass; P_area_, leaf phosphorus per unit area; K_mass_, leaf potassium per unit mass; K_area_, leaf potassium per unit area.

### The final SEM model for P_mass_

**Fig 2:**
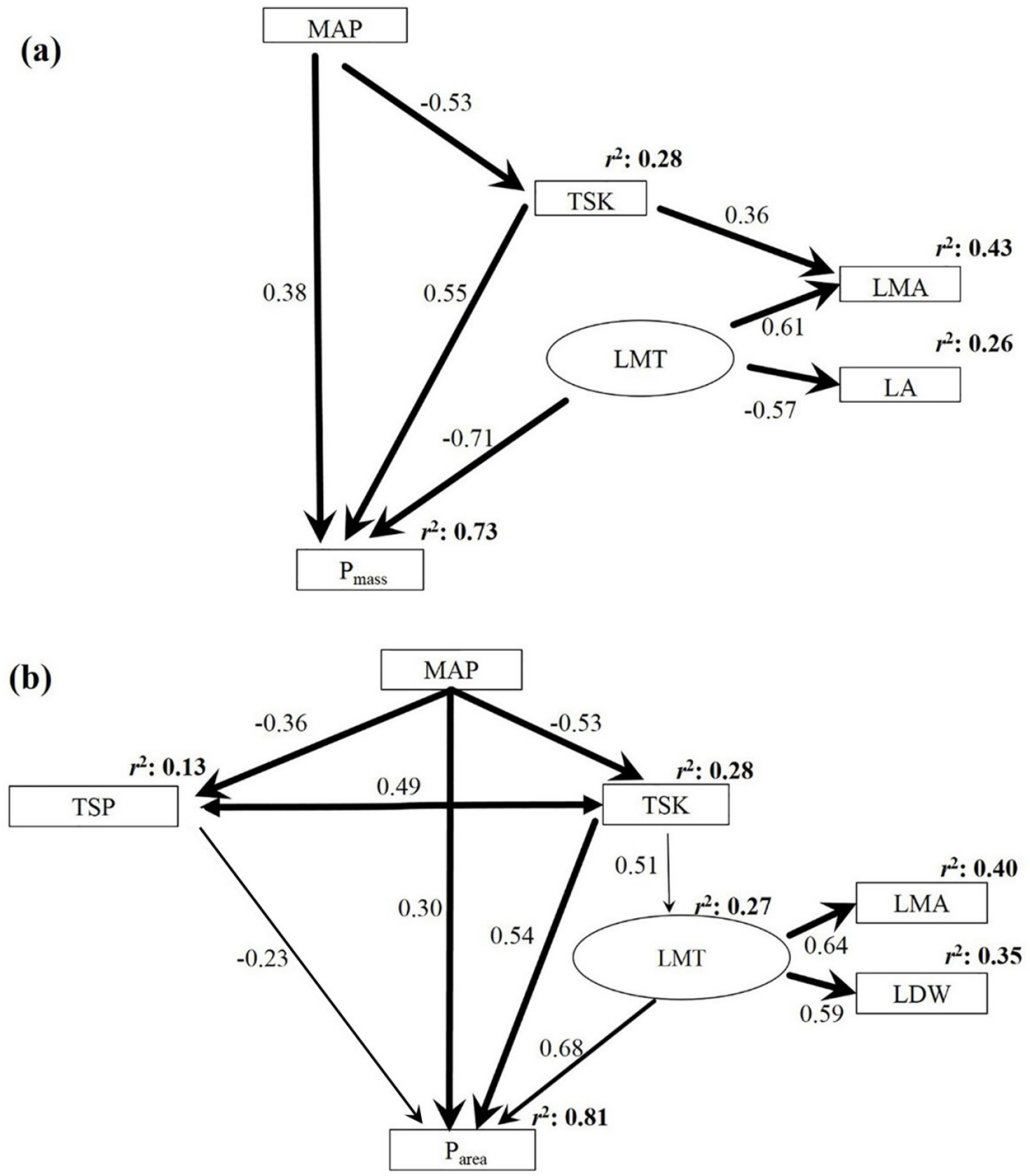
Final SEMs linking P_mass_ and P_area_ and their drivers. Single headed arrows indicate a causal influence of one variable upon another. Narrow arrows indicate *P* < 0.05; wider arrows indicate *P* < 0.01; and the widest arrows indicate *P* < 0.001. Values on arrows indicate standardized coefficients. The value at the top-right corner of each variable (*r*^2^) represents the proportion of variance explained. Abbreviations: MAP, mean annual precipitation; TSK, total soil potassium; TSP, total soil phosphorus; LMT, leaf morphological trait; LMA, leaf mass per area; LA, leaf area; LDW, leaf dry weight; P_mass_, leaf phosphorus per unit mass; P_area_, leaf phosphorus per unit area.

The final SEM model for P_mass_ had a good fit to the data, and explained 73% of the variation in P_mass_ (Tables 3 and 4, Fig 2a). The model revealed that P_mass_ was impacted by leaf morphological traits (including LMA and LA), MAP, and TSK. Leaf morphological traits had the largest standardized total effect on P_mass_ (-0.71) among all factors (Table 4, Fig 2a). Increasing TSK led to an increase in P_mass_ (0.55). MAP had a positive direct effect P_mass_, but a negative indirect effect via TSK (Table 4, Fig 2a). As the direct and indirect effects of MAP on P_mass_ were approximately the same (but in opposite directions), MAP had a limited total effect (0.09) on P_mass_ (Table 4).

**Table 3:** Structural equation model fit indices and evaluation criteria.

Indices	Evaluation criteria or critical value for fit	P_mass_	P_area_
**χ**^**2**^	*P* > 0.05	χ^2^ = 2.886 *P* = 0.410	χ^2^ = 5.162 *P* = 0.396
**RMSEA**	< 0.08	0.000	0.022
**NC**	< 3	0.946	1.034
**AGFI**	> 0.9	0.919	0.902
**NFI**	> 0.9	0.968	0.965

χ^2^, the chi-square test; RMSEA, the root square mean error of approximation; AGFI, the adjusted goodness of fit index; NFI, the normed fit index; NC, χ^2^ divided by its degrees of freedom; P_mass_, leaf P concentration per unit mass; P_area_, leaf P concentration per unit area.

**Table 4:** Paths and standardized effects each predictor for foliar P.

Model	Predictor	Paths	Standardized effect
P_mass_	MAP	Direct	0.38***
		Indirect through TSK	-0.29***
		Total	0.09
	TSK	Direct	0.55***
		Indirect	--
		Total	0.55
	LMT (LMA and LA)	Direct	-0.71***
		Indirect	--
		Total	-0.71
P_area_	MAP	Direct	0.30***
		Indirect through TSK	-0.47**
		Indirect through TSP	0.08*
		Total	-0.09
	TSK	Direct	0.54***
		Indirect through LMT	0.35**
		Total	0.89
	TSP	Direct	-0.23*
		Indirect	--
		Total	-0.23
	LMT (LMA and LDW)	Direct	0.68**
		Indirect	--
		Total	0.68

Significant effects are at *P* < 0.05 (*), < 0.01 (**), and < 0.001 (***). MAP, mean annual precipitation; TSK, total soil potassium; TSP, total soil phosphorus; LMT, leaf morphological trait; LMA, leaf mass per area; LA, leaf area; LDW, leaf dry weight; P_mass_, leaf phosphorus per unit mass; P_area_, leaf phosphorus per unit area.

### The final SEM model for P_area_

The final SEM model for P_area_ also fit well with the data, and explained 81% of the variation in P_area_ (Tables 3 and 4, Fig 2b). P_area_ was influenced by leaf morphological traits (including LMA and LDW), MAP, TSK, and TSP. Leaf morphological traits had an effect on P_area_ (0.68) (Table 4, Fig 2b). MAP had a positive direct effect (0.30), a negative indirect effect via TSK (-0.47), and a positive indirect effect via TSP (0.08), resulting in a total effect of -0.09 on P_area_ (Table 4). TSK had a positive direct (0.54) and a positive indirect effect through leaf morphological traits (0.35) on P_area_. TSP had a negative direct effect P_area_ (-0.23), and was positively correlated with TSK (0.49) (Table 4, Fig 2b).

## Discussion

To the best of our knowledge, this study represents the first attempt to describe intraspecific leaf P variation across a large geographic scale. Meanwhile, mean value of leaf N:P ratio exceeding the Redfield ratio suggested the P limited status (N:P ratio = 16) of our research subjects [13, 54], which made our research on the primary drivers on leaf P more necessary. Directly comparing the significant correlations of total soil potassium and leaf P and non-significant correlations of total soil phosphorus and leaf P, we were naturally impressed by the larger role of soil K in determining leaf P instead of soil P, meanwhile multiple determinants of the leaf phosphorus status can provide us more evidences. Using structural equation models, we discovered that the P_mass_ and P_area_ of *Q*. *wutaishanica* in Northern China were collectively influenced by leaf morphological traits, mean annual precipitation, and soil nutrients. These complex P_mass_ and P_area_ associated causal relationships would be difficult to obtain from small-scale experiments, or empirical bivariate studies.

### Characteristics of the leaf P_mass_ and P_area_ variation in *Q*. *wutaishanica* across the Loess Plateau

We found that P_mass_ and P_area_ were linked with leaf morphological traits. Consistent with previous interspecific findings [33, 34], LMA was negatively correlated to P_mass_, and positively correlated to P_area_. Across plant species, leaf assimilation capacity per unit area positively correlated to N_area_ and P_area_ and increased with increasing LMA [34]. By contrast, the foliage assimilation capacity per unit mass positively correlated to N_mass_ and P_mass_ [17], and scaled negatively with LMA [29]. The positive correlation between LMA and P_area_ found in *Q*. *wutaishanica* suggested that leaves of higher LMA enhanced the quantity of photosynthetic tissues per unit area, similar to the relationship between LMA and N_area_ across species from different habitats [29, 34]. Increases in LMA prompt the enhancement of the intercellular transfer resistance to CO_2_, and decreases in assimilative leaf compounds, thus leading to the negative correlation between LMA and P_mass_, similar to the relationship between LMA and N_mass_ [29, 34]. Collectively, the relationships between foliar P content and LMA in *Q*. *wutaishanica* were similar to those reported across a range of species [34].

### Relationship between leaf P content and leaf morphological traits

With the influences of LMA simultaneously accounted for in our structural equation models, we found, in agreement with our hypothesis, that there were also positive correlations between LA and P_mass_, and between LDW and P_area_. The negative correlation between LMA and LA was similar to the results of *Q*. *ilex* [55], indicating a reduction in support tissue per unit area as the leaf area increases within species. Higher P_mass_, and thus higher net photosynthetic rate per mass [54] in larger leaves appears to be an adaptation of lower LMA [33]. The positive correlation between LA and P_mass_ may have resulted from the positive effects of leaf P concentration on leaf expansion rates, and the increased production of assimilates required for growth over the duration of expansion [36].

### Important effects of both total soil K and P on leaf P content according to the SEMs

We revealed positive effects of total soil K on P_mass_, and P_area_. The positive effects of soil K on both P_mass_ and P_area_ of *Q*. *wutaishanica* from direct and indirect paths supported the hypothesis that soil K plays critical roles in increasing foliar P. Although leaves of K-deficient plants accumulate sugars, they rarely increase their root biomass, because they are less able to translocate sucrose to the root via the phloem [24], which decreases root and/or mycorrhizal fungi biomass for the absorption of P [25, 27]. These processes may be responsible for the positive effects of soil K both P_mass_ and P_area_ we observed in the system with low environmental availability of inorganic P [8, 15].

We found no significant effects of soil resident P on foliar P_mass_, but a negative direct effect of soil P on P_area_, while soil N had little effects on either P_mass_ or P_area_. Typically, plant and soil P are coupled [54, 56], and the P_mass_ of 753 terrestrial plant species increased significantly with increasing soil P in China [8]. The lack of the effect of soil P on P_mass_ in our study may be attributed to that photosynthetic products that are prioritized to meet requirements for the growth of roots rather than leaves in low soil P habitats to ensure a necessary supply of P [4, 24, 52, 54, 56–57]. It means that coupled change between leaf P supply and photosynthetic production allocated to leaves lead to the non-significant correlation between P_mass_ and soil P, even though soil P of more than 6-fold was reported here. Although our bivariate analysis showed a positive effect of TSP on P_area_, similar to those reported previously [34, 58], our SEM analysis indicates that the direct effect of TSP on P_area_ was negative, but complimented by the positive effect of TSK on P_area_.

These complex relationships likely involve several mechanisms. In a P limited environment, plants reduce disparities in the supply of photosynthetic products and nutrients by enhancing their capacity to acquire the most limiting resource [59], leading to the allocation of additional photosynthetic products to root systems and less to leaf biomass. Higher plant nutrient concentrations in nutrient-poor environments enhanced their competitive capacity associated with plant growth capacity[16], meanwhile, plants increase leaf thickness in order to cope with low soil P [29]. Therefore, the negative direct effect of soil P on P_area_ likely reflects the collective influence of reduced photosynthetic product supplies to leaves and increased leaf thickness for more proportion of mesophyll tissue [29]. Conversely, increasing soil P associated with increasing soil K, due to their simultaneous provision from weathering of parent materials and uplift, and release at the surface soil layer by plants through litter fall and fine root turnover [44, 45] impart a positive effect on P_area_. Unlike the overwhelmingly demonstrated positive effects of P addition on P_mass_ [11], little is known about the P addition effects on P_area_. To better elucidate the influence of soil P on P_area_, future methodical experiments may further verify the complex mechanisms that we observed in our study.

### Different paths leading to the combined effects of precipitation on leaf P content

In contrast to regional and global level reports [7, 17], mean annual temperature had no effect on leaf traits in our study (which might have resulted from the limited temperature variation in our sample set), and was thus excluded from our SEMs. Consistent with Ordoñez, Van Bodegom (20) and Maire, Wright (34), the variation of P_mass_ and P_area_ that could be explained by mean annual precipitation was exacted but modest. Nonetheless, the direct positive and indirect negative effects of precipitation on P_mass_ and P_area_ were all of significance. This key environmental variable should not be simply ignored due to the different mechanisms involved with direct and indirect effects. Forest sites that receive higher precipitation are typically characterized by the augmented downward transport of humic substances in the soil profile, and enhanced transmission of nutrients within the rhizosphere [60], which might assist with increasing the P supply for *Q*. *wutaishanica*. Nevertheless, the indirect effect of precipitation on foliar P content, mediated via TSK, was negative. This was likely due to soil P and K leaching losses, which increased in conjunction with precipitation [22, 23].

Our study revealed the variation of P_mass_ and P_area_ in *Quercus wutaishanica* leaves along with multiple factors in the Loess Plateau, China. Consistent with previous interspecific findings, we revealed that leaf mass per area was negatively correlated with P_mass_, and positively correlated with P_area_. We also observed positive correlations between leaf dry weight and P_area_, and between leaf area and P_mass_. We found that total soil K was the most critical environmental driver for leaf P, whereas the total P concentration of the soil had no effect on P_mass_. Moreover, our SEM highlighted a direct negative effect of total soil P on P_area_, and indicated that the positive bivariate correlation between total soil P and P_area_ masked the direct negative effect of total soil P, and positive effect of total soil K on P_area_. Future nutrient addition experiments might facilitate the testing of mechanistic links between soil P, soil K, and leaf P, particularly P_area_, by simultaneously examining above- and belowground nutrient allocation and leaf morphological acclimation, meanwhile researches focusing on the mechanism of soil K in determining leaf P and accurate proportion of leaf K, P taken up from different soil layers have important implications in understanding the forest potassium and phosphorus cycling.

## Supporting information

S1 Table Major climates and geographic information of each sampling sites. MAT, mean annual temperature; MAP, mean annual precipitation.
(DOCX)
