# Supplementary material for: Leaf phosphorus content of Quercus wutaishanica increases with total soil potassium in the Loess Plateau
Source: PLoS One. 2018 Aug 2;13(8):e0201350. doi: 10.1371/journal.pone.0201350 (PMC6072014; doi:10.1371/journal.pone.0201350)
Supplement: S1 Table — MAT, mean annual temperature; MAP, mean annual precipitation. (DOCX) [file pone.0201350.s001.docx]

**Table S**1 Maj**or climates and geographic information of each sampling sites.**

| **Plots** | **Mountains** | **MAT (℃)** | **MAP (mm)** | **Latitude (°)** | **Longitude (°)** | **Elevation (m)** |
| --- | --- | --- | --- | --- | --- | --- |
| **1** | Ziwuling | 10.3 | 554 | 35.65243 | 108.6851 | 1252 |
| **2** | Ziwuling | 9.9 | 621 | 35.09806 | 108.648 | 1295 |
| **3** | Ziwuling | 9.4 | 564 | 35.65905 | 108.6073 | 1310 |
| **4** | Ziwuling | 9.1 | 566 | 35.66199 | 108.5535 | 1400 |
| **5** | Ziwuling | 8.9 | 568 | 35.65954 | 108.5467 | 1510 |
| **6** | Ziwuling | 8.4 | 574 | 35.65014 | 108.5269 | 1590 |
| **7** | Taiyue | 6.4 | 588 | 36.61351 | 111.9938 | 1478 |
| **8** | Taiyue | 6.3 | 603 | 36.40922 | 111.8492 | 1525 |
| **9** | Taiyue | 6 | 608 | 36.40589 | 111.8495 | 1616 |
| **10** | Taiyue | 5.1 | 623 | 36.41972 | 111.8695 | 1708 |
| **11** | Taiyue | 4.8 | 629 | 36.43128 | 111.8798 | 1808 |
| **12** | Taiyue | 4.9 | 626 | 36.43451 | 111.8792 | 1810 |
| **13** | Taiyue | 4.9 | 626 | 36.44059 | 111.8803 | 1914 |
| **14** | Taiyue | 4.8 | 628 | 36.43596 | 111.8744 | 2015 |
| **15** | Taiyue | 4.8 | 628 | 36.44122 | 111.8673 | 2115 |
| **16** | Taiyue | 4.8 | 628 | 36.43976 | 111.8685 | 2222 |
| **17** | Taihang | 5 | 642 | 37.10868 | 113.476 | 1509 |
| **18** | Taihang | 5 | 642 | 37.11068 | 113.4819 | 1608 |
| **19** | Taihang | 5.2 | 635 | 37.13273 | 113.5018 | 1707 |
| **20** | Taihang | 4.1 | 660 | 37.13302 | 113.4905 | 1918 |
| **21** | Taibai | 6.8 | 855 | 34.05624 | 107.6999 | 2000 |
| **22** | Taibai | 6.5 | 864 | 34.04959 | 107.7061 | 2136 |
| **23** | Taibai | 6.5 | 864 | 34.05247 | 107.7059 | 2231 |
| **24** | Taibai | 5.3 | 889 | 34.05506 | 107.7038 | 2303 |
| **25** | Huanglong | 8.8 | 577 | 35.61048 | 109.9475 | 1330 |
| **26** | Huanglong | 8.4 | 577 | 35.62603 | 109.9699 | 1400 |
| **27** | Huanglong | 7.7 | 581 | 35.64889 | 109.9945 | 1530 |
| **28** | Huanglong | 7.4 | 581 | 35.66684 | 110.0199 | 1700 |
| **29** | Guan | 7.8 | 678 | 34.73258 | 106.6833 | 1704 |
| **30** | Guan | 7.4 | 688 | 34.71136 | 106.6823 | 1800 |

MAT, mean annual temperature; MAP, mean annual precipitation.
